# Supplementary material for: Complete reference genome and pangenome improve genome-wide detection and interpretation of DNA methylation using sequencing and array data
Source: Cell Rep. Author manuscript; Available in PMC 2025 Jul 24. (PMC12288590; doi:10.1016/j.celrep.2025.115755)
Supplement: 1 [file NIHMS2092646-supplement-1.pdf]

Cell Reports, Volume *44*

Supplemental information

Complete reference genome and pangenome improve genome-wide detection and interpretation of DNA methylation using sequencing and array data

Zheng Dong, Joanne Whitehead, Maggie Fu, Julia L. MacIsaac, David H. Rehkopf, Luis Rosero-Bixby, Michael S. Kobor, and Keegan Korthauer

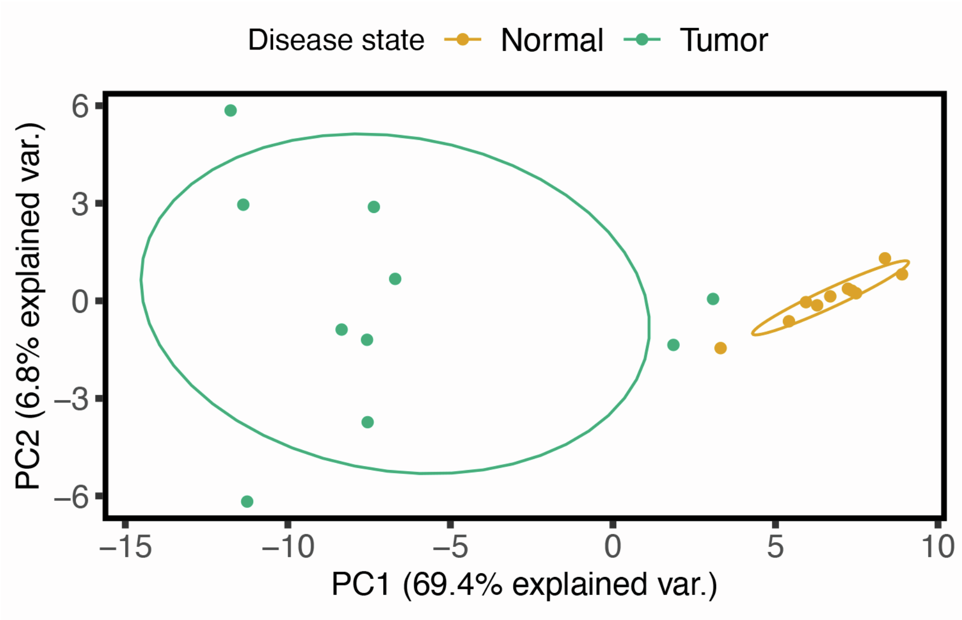

Figure S1. Loadings using the first two principal components (PCs) for each RRBS sample resulting from PCA color-coded by disease state of colon cancer.

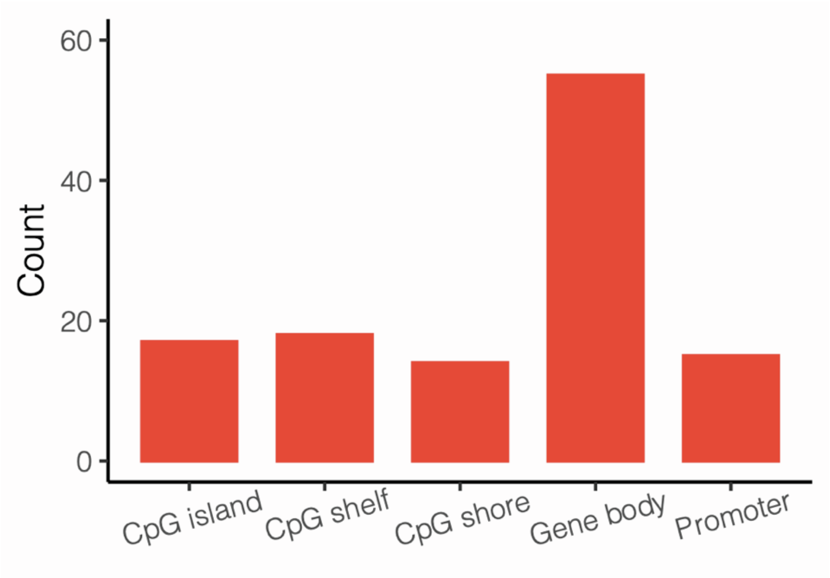

Figure S2. Colocalization of colon cancer-associated CpGs that benefited from the additional CpGs called using T2T-CHM13 (n = 88) with genomic elements. Some CpGs overlapped multiple genomic features and were counted for each feature independently.

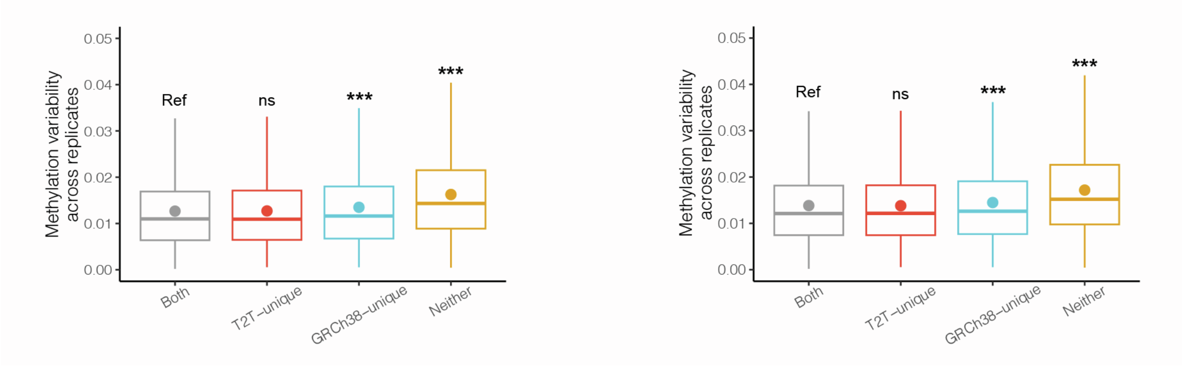

Figure S3. DNAm variability of CpGs in HM450K (left) and EPIC (right) across one blood sample with six technical replicates in all CpG groups. Mean values are represented as circles in each box plot. Not significant (ns); FDR ≥ 0.05; * 0.01 ≤ FDR < 0.05; ** 0.001 ≤ FDR < 0.01; *** FDR < 0.001. Outliers are not shown in box plots.

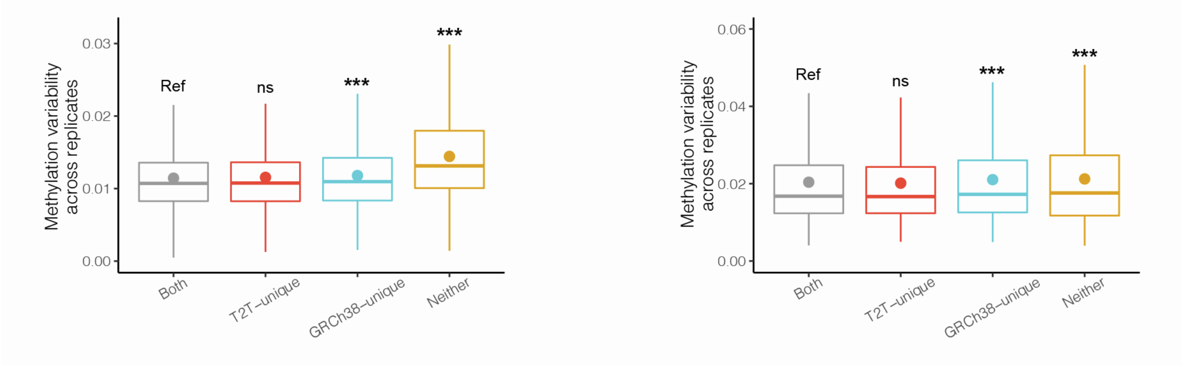

Figure S4. DNAm variability of HM450K CpGs across 36 blood (left) and cerebellum (right) samples with two technical replicates each for all CpG groups. Mean values are represented as circles in each box plot. Not significant (ns); FDR ≥ 0.05; * 0.01 ≤ FDR < 0.05; ** 0.001 ≤ FDR < 0.01; *** FDR < 0.001. Outliers are not shown in box plots.

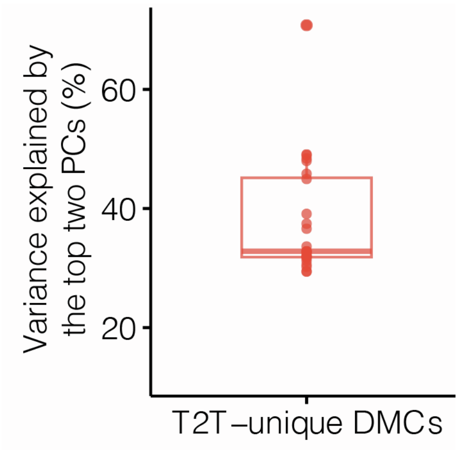

Figure S5. Boxplots illustrating the proportions of variance explained by the top two PCs for DMCs found only with T2T-CHM13 in various cancers. Each dot represents a type of cancer.

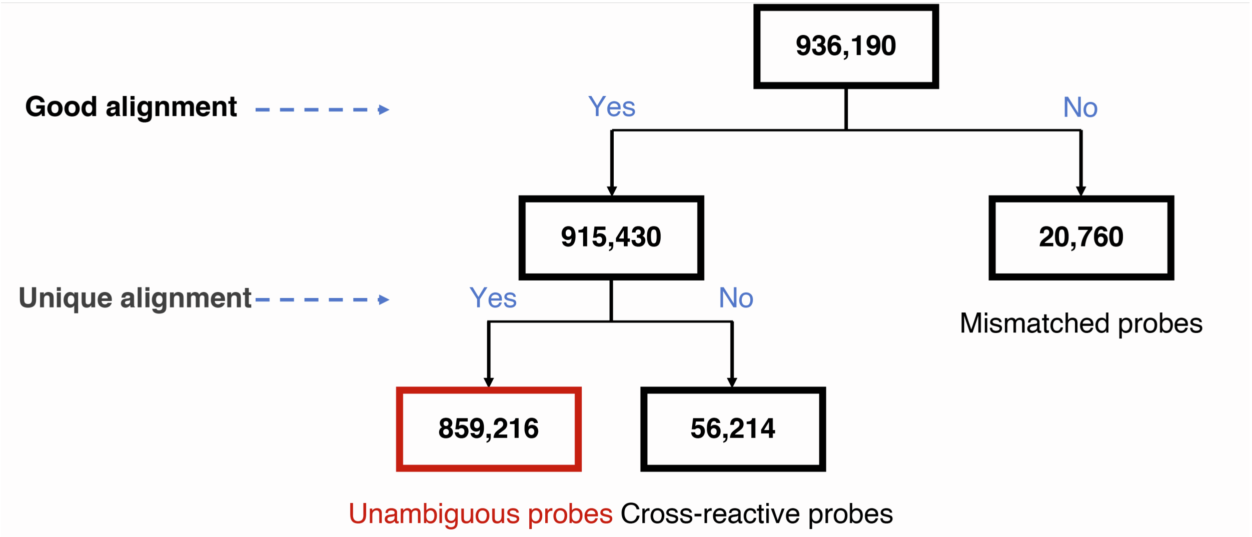

Figure S6. Flowchart of unambiguous probe discovery in the EPICv2 array using T2T-CHM13. Probes in the array were first aligned to the reference genome to determine whether they showed good alignment (i.e., sequence alignment with at least 90% identity, at least 40 of 50 matching bases, no gaps, and the CpG locus had to be perfectly matched); if not, they were classified as mismatched probes; if yes, they were further checked to determine whether they were unique alignments; if yes, they were classified as unambiguous probes (i.e., non–cross-

reactive and non-mismatched probes uniquely mapping to the target region); otherwise, they were classified as cross-reactive probes.

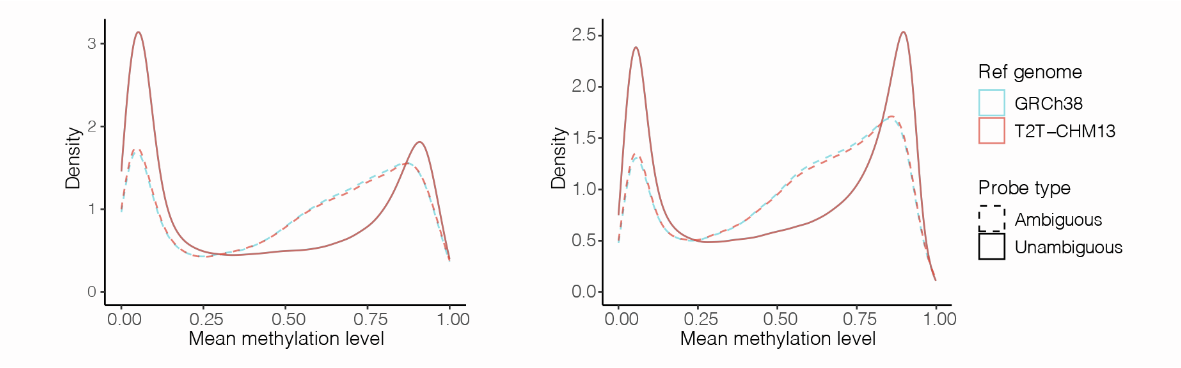

Figure S7. Mean methylation density differences between ambiguous and unambiguous probes in HM450K (left, *n* = 4) and EPIC (right, *n* = 3) across technical replicates from the IMR-90 cell line. Ambiguous probes were defined as those with cross-reactivity and/or mismatch.
